# Self-assembled monolayers of pyridylthio-functionalized carbon nanotubes used as a support to immobilize cytochrome c

**DOI:** 10.1186/1556-276X-8-63

**Published:** 2013-02-07

**Authors:** Qing Sun, Jiang Liu, Hong-Xiang Huang, Meng Chen, Dong-Jin Qian

**Affiliations:** 1 Department of Chemistry, Fudan University, 220 Handan Road, Shanghai, 200433, China; 2 Department of Macromolecular Science, Fudan University, 220 Handan Road, Shanghai, 200433, China

**Keywords:** Carbon nanotube, Self-assembled monolayer, Protein, Adsorption, Morphology

## Abstract

Self-assembled monolayers (SAMs) of pyridylthio-functionalized multiwalled carbon nanotubes (pythio-MWNTs) have been constructed on the gold substrate surface, which were used as a support to immobilize cytochrome c (Cyt c). The assembly processes of the SAMs and adsorption of Cyt c were monitored by using quartz crystal microbalance (QCM). Based on the frequency change of the QCM resonator, the surface coverage for the SAMs of pythio-MWNTs was estimated to be about 5.2 μg/cm^2^, and that of the Cyt c adsorbed was about 0.29 μg/cm^2^. For the gold electrode modified by the SAMs of pythio-MWNTs-Cyt c, a quasi-reversible redox wave was recorded with the cathodic and anodic potentials at about −0.55 and −0.28 V vs Ag/AgCl, respectively. Compositions and morphologies of the SAMs before and after immobilization of Cyt c were characterized by X-ray photoelectron spectroscopy, Raman spectroscopy, scanning electron microscopy, and atomic force microscopy.

## Background

Self-assembly of a molecular monolayer or nanopatterns onto a solid surface has attracted much attention because of important academic researches and a wide variety of potential applications such as adhesion, lubrication, corrosion inhibition, and micro-/nanoelectronic devices [1-3]. Many organic compounds and nanomaterials have been anchored on the gold surface through the sulfur (thiol, disulfide, or thioether) groups or on the quartz and glass surfaces through the siloxane linkage [4,5]. Both of them provide strong interaction at interfaces, which results in an easy construction of well-defined self-assembled monolayers (SAMs). These SAMs are highly ordered two-dimensional (2D) monolayers with densely packed molecular arrangement and controllable structural regularity. When suitable or desired molecules or nanomaterials are used, the as-prepared SAMs can act as a 2D support to react with other functional materials for the fabrication of (bio)sensors, artificial light-harvesting units to mimic energy transfer processes or act as heterogeneous catalysts, and so on [6-8].

Carbon nanotubes (CNTs) possess unique mechanical, thermal, and electrical properties that suggest a wide range of applications in the fields of new materials and nanotechnology [9]. One kind of very often investigated new materials is prepared via an intermolecularly covalent or noncovalent interaction between CNTs and organic or polymeric species, resulting in the formation of novel CNT-containing nanocomposites or nanohybrids with improved solubility or suspensions in liquids as well as new functions [10,11]. For instance, the oxidized CNTs have been widely used to bind with polyelectrolytes or proteins to produce new hybrid materials based on the molecular electrostatic interaction, which have the functions of both CNTs and polyelectrolytes or proteins [12,13]. These oxidized CNTs can also react with the amino substituents of proteins for the formation of CNT-protein nanocomposites [14,15].

In the present work, the oxidized multiwalled CNTs (MWNTs) were reacted with *S*-(2-aminoethylthio)-2-thiopyridine hydrochloride to form pyridylthio-modified MWNT (pythio-MWNT) nanohybrids according to You et al.'s method [16]. These pythio-MWNTs are considered to have the following features: (1) increased solubility in organic solvents; (2) possible reaction with proteins via the S-S bond, except for the adsorption on the surface of pythio-MWNTs [16,17]; and (3) possible construction of SAMs on the gold electrode surface via the Au-S bond. We have previously reported that these pythio-MWNT hybrids could form stable Langmuir-Blodgett (LB) films, which acted as a support to immobilize hydrogenase (H_2_ase) [17]. The as-prepared LB films of pythio-MWNTs-H_2_ase showed strong stability in solutions and higher bioactivity compared with those ordered aggregates formed with polyelectrolytes. Here, the SAMs of pythio-MWNT hybrids were constructed on the gold surface and used as a support to immobilize cytochrome c (Cyt c). The assembly process of SAMs and adsorption of Cyt c were characterized by using quartz crystal microbalance (QCM), Raman spectroscopy, X-ray photoelectron spectroscopy (XPS), scanning electron microscopy (SEM), and atomic force microscopy (AFM).

## Methods

### Materials

Multiwalled carbon nanotubes (diameter, 3~10 nm) were purchased from Strem Chemicals (Newburyport, MA, USA). Cytochrome c, 1-[3-(dimethylamino)propyl]-3-ethylcarbodiimide hydrochloride (DEC), aldrithiol-2, and 2-aminoethylthiol hydrochloride were from Sigma-Aldrich Co. (St. Louis , MO, USA). *NN*′-dimethylformamide (DMF) was from Fisher Scientific Co. (Hampton, NH, USA). All chemicals were used as received without further purification. *S*-(2-aminoethylthio)-2-thiopyridine (AETTPy) was synthesized according to the method described by You and coworkers [16] and checked by ^1^HNMR and elemental analysis [17]. Ultrapure water (18.2 MΩ cm) for the subphases was prepared with a Rephile filtration unit (Rephile Bioscience Ltd., Shanghai, China).

### Functionalization of carbon nanotubes

The as-received MWNTs were firstly oxidized using an acid oxidative method [18] and then reacted with AETTPy [16]. The produced pythio-MWNT nanohybrids were collected by centrifugation, washed well with water to remove unreacted reactants, and finally dried in vacuum. The obtained solid sample of pythio-MWNTs was analyzed by elemental and thermogravimetric analyses as described in our previous work [17].

### Self-assembled monolayers

Pythio-MWNT nanohybrids were anchored on the surface of AT-cut gold-coated quartz crystals for the QCM and XPS measurements as well as for the morphology characterization. The resonant frequency of the crystals was 9 MHz (5 mm in diameter, Seiko EG&G, Seiko Instruments Inc., Chiba, Japan). The frequency of the QCM was measured with a Seiko EG&G model 917 quartz crystal analyzer. The crystal was mounted in a cell by means of O-ring seals, with only one face in contact with the solution. Before assembly, the crystal was cleaned in a piranha solution (H_2_SO_4_/H_2_O_2_; 3:1) for 10 min, then washed with a copious amount of water, and finally dried and kept under Ar atmosphere.

After pretreatment, the QCM crystals were immersed in the DMF solution containing 2 mg/ml pythio-MWNT nanohybrids for 2 days, which were then rinsed with a copious amount of ethanol and water. The frequency before and after assembly was measured for the estimation of the amount of the nanohybrids anchored on the gold surface. For the immobilization of Cyt c, the as-prepared pythio-MWNT SAMs were immersed in the QCM cell containing 2 mg/ml Cyt c. The frequency was recorded after the modified quartz crystal was immersed in the solution.

### Instruments

XPS spectra were recorded using a VG ESCALAB MKII multifunction spectrometer (VG Scientific, East Grinstead, West Sussex, UK), with nonmonochromatized Mg-Kα X-rays as the excitation source. The system was carefully calibrated by the Fermi edge of nickel and the Au 4*f*_2/7_ and Cu 2*p*_2/3_ binding energies. A pass energy of 70 eV and a step size of 1 eV were chosen when taking spectra. In the analysis chamber, pressures of 1~2 × 10^−7^ Pa were routinely maintained. The binding energies obtained in the XPS analysis were corrected by referencing the C1*s* peak to 284.60 eV.

Raman spectra were recorded on an SPEX 1403 spectrometer (SPEX Industries, Inc., Edison, NJ, USA) and excited at 633 nm by a He-Ne laser. SEM images of the SAMs were observed on a Philips XL30 electron microscope (FEI Co., Hillsboro, OR, USA). AFM images were observed using an SPM-9500J3 scanning probe microscope (Shimadzu Corporation, Kyoto, Japan). Tapping mode was used with a tip fabricated from silicon (130 μm in length with *ca.* 40 kHz resonant frequency) in air. In all cases, the SAMs of pythio-MWNTs and their nanocomposites with Cyt c were assembled on freshly prepared gold substrate surfaces.

Cyclic voltammogram was measured using an electrochemical analyzer (CHI 601b, CH Instruments, Inc., Shanghai, China). A Pt wire and Ag/AgCl electrode were used as the auxiliary and reference electrodes, respectively, and the Au electrode covered with the SAMs of pythio-MWNTs-Cyt c was used as the working electrode with 0.01 mol/l KCl as the electrolyte. An initial potential of 0.2 V was applied for 2 s, and subsequently, cyclic scans to a final potential of −0.8 V were done for 10 cycles. All electrochemical measurements were done under an Ar atmosphere at room temperature.

## Results and discussion

### Construction of self-assembled monolayers and QCM response

**Figure 1:**
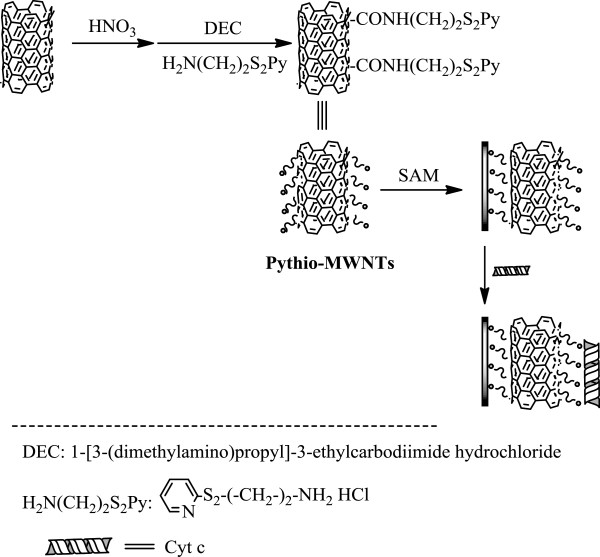
Schematic drawing of the pythio-MWNT SAMs and adsorption of Cyt c.

Figure 1 shows a schematic representation for the synthesis of the linkage of AETTPy, functionalization of the MWNT nanohybrids, assembly of the pythio-MWNT SAMs, as well as formation of the nanocomposites with the protein on the gold surface. Details on the elemental and thermogravimetric analysis of AETTPy and pythio-MWNT hybrids have been described previously [17]. Here, the as-prepared pythio-MWNTs were ultrasonically dissolved in DMF, the solution of which was centrifuged to remove ‘undissolved’ solid powders. A freshly prepared QCM crystal or gold substrate was immersed in the dark solution of pythio-MWNTs for the formation of the SAMs, which was then immersed in the Cyt c solution for the formation of the bio-nanocomposites. The assembly or adsorption process was monitored by measuring the frequency change of the QCM resonator. 

Generally, the assembly of organic molecules such as viologenthiol derivatives on the gold surface could be completed within several hours [19,20]. During the experiments, we found that formation of the present pythio-MWNT SAMs took quite a long time (over 10 h); thus, we measured the frequency change of the QCM resonators before and after the assembly instead of recording the whole dynamic assembling process. A possible reason for such a slow assembly was the fact that the pythio-MWNT hybrids were nanomaterials with a ‘molecular weight’ much larger than that of the commonly used organic molecules; thus, both the Au-S bond formation and ‘molecules’ (pythio-MWNT hybrids) moving in the solution were very slow.

The frequency change (Δ*F*) was about 4.88 kHz after formation of the pythio-MWNT SAMs. Based on the equation of *ΔF* = −2*F*_0_ ^2^*Δm*/(*A ρ*_q_ ^1/2^*μ*_q_ ^1/2^), where *F*_0_ is the fundamental resonant frequency (9 MHz), Δ*m* (g) is the mass change, *A* is the surface area (0.196 cm^2^) of the QCM resonator, *ρ*_q_ is the density of the quartz (2.65 g/cm^3^), and *μ*_q_ is the shear module (2.95 × 10^11^ dyne/cm^2^) [21], the mass change was about 5.2 μg/cm^2^.

**Figure 2:**
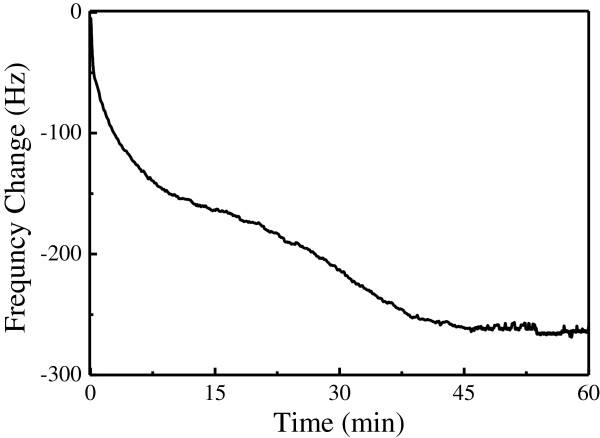
Frequency change with adsorption time for the pythio-MWNTs SAMs in the Cyt c solution.

After composition and morphology characterization of the pythio-MWNT SAMs (as to be described below), the SAMs were immersed in the Cyt c solution to form pythio-MWNT-Cyt c bio-nanocomposites, the adsorption process of which was also monitored by using QCM. Figure 2 shows the frequency change Δ*F* as a function of time (*t*) for the SAMs of pythio-MWNTs immersed in the 2 mg/ml solution Cyt c. The curve indicated that the frequency decreased quickly at the initial 10 min, then this decrease became slower and slower (a platform-like stage was observed). After about 40 min, the frequency did not show an obvious decrease, and a platform was formed.

This Δ*Ft* curve suggested that adsorption of the Cyt c on the SAMs of pythio-MWNTs was very quick at the initial 10 min and then became slower to reach an equilibrium state between adsorption and desorption. The whole assembly could be completed within 1 h. During the adsorption of the proteins on the surface of the SAMs, a platform-like stage may indicate that the adsorption was very quick at the ‘naked’ SAM surface. Then, two processes may dominate the adsorption: one was the equilibrium state between adsorption and desorption and the other one may be the formation of double layers. Based on the Δ*F* value, we calculated that the amount of the Cyt c adsorbed was about 0.29 μg/cm^2^. Since the molecular weight of Cyt c was about 11,000~13,000, the surface density of the Cyt c was about 0.22~0.26 × 10^−10^ mol/cm^2^. Kutner and coworkers reported that the Cyt c adsorbed on the fullerene film-modified electrode was in the range of 0.5~2.5 × 10^−10^ mol/cm^2^[22], which was in agreement with that observed in the present work.

### X-ray photoelectron and Raman spectroscopy

**Figure 3:**
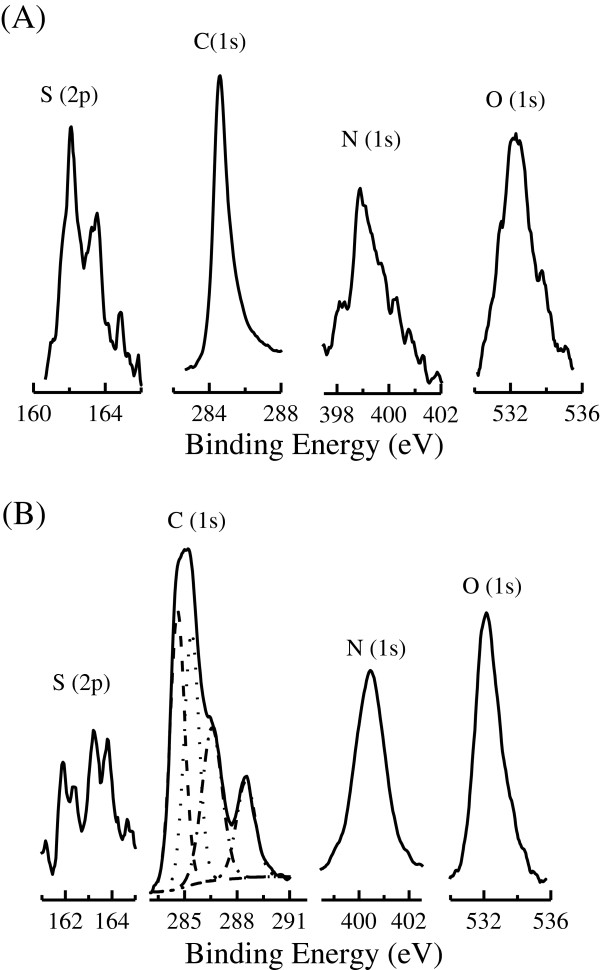
**XPS spectra.** (**A**) SAMs of pythio-MWNTs and (**B**) nanocomposites of pythio-MWNTs-Cyt c.

Element compositions for the SAMs of pythio-MWNTs before and after adsorption of Cyt c were detected using the XPS spectra, which revealed four peaks in the binding energy from 100 to 600 eV except for the Au from the substrate surface. As shown in Figure 3A, the binding energies for these four peaks were as follows: 162.1~164.8, 284.6, 398.9, and 532.3 eV, which could be assigned to the elements of S(2*p*), C(1*s*), N(1*s*), and O(1*s*), respectively. The binding energies for these elements in the powders of pythio-MWNTs were 164.3~165.6, 284.8, 399.4, and 532.4 eV, respectively (figures not shown), which were in agreement with those in the SAMs. The C (partly) and O elements were from carbon nanotubes, while the elements of S, N, and C (partly) were from the functionalized pythio-substituents (AETTPy) of the nanohybrids. Thus, these XPS data confirmed that the SAMs of pythio-MWNTs have been formed on the gold surface.

Figure 3B shows the highly resolved XPS spectra of the pythio-MWNTs after being immersed in the Cyt c, which also revealed four groups of peaks corresponding to the elements of S, C, N, and O. A close inspection of the spectra could find that the C(1*s*) spectrum was composed of several peaks in the binding energy range from 285 to 290 eV. Shim and coworkers recently prepared biomimetic layers of Cyt c. They reported that when the Cyt c was adsorbed on the Langmuir-Blodgett films of the polymer nanocomposites, there was a broad band at around 287.6 eV corresponding to the C=O, C-O, or O-C-O substituents [23]. Here, the binding energy of the C element appeared at about 285.1, 286.6, and 288.5 eV. The different feature for the binding energy of the C element could be attributed to the adsorbed Cyt c. Other elements of S, N, and O showed the binding energy at about 161.9~163.8, 400.4, and 532.2 eV, which was in agreement with that in the SAMs of pythio-MWNTs.

A comparison for the peaks of S(2*p*) and N(1*s*) before and after the adsorption of Cyt c could further find the following two features. The first one was that the binding energy of S(2*p*) slightly shifted after the adsorption, which may be attributed to the formation of the Au-S bond in the SAMs of pythio-MWNTs. The second one was that the maximum binding energy of N(1*s*) atoms shifted from 398.9 to 400.4 eV, which may be designated to the contribution of N atoms in the Cyt c together with that in the SAMs.

**Figure 4:**
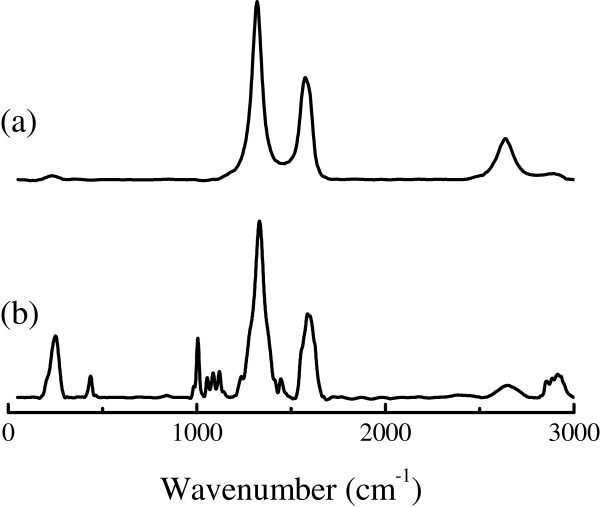
**Raman spectra.** (**a**) Commercial MWNTs and (**b**) SAMs of pythio-MWNTs.

Figure 4 shows the Raman spectra for the commercial MWNTs, and SAMs of pythio-MWNT nanohybrids. Two separated peaks were recorded for the commercial MWNTs and appeared at about 1,320 and 1,574 cm^−1^. It has been pointed out that the peak at about 1,330 cm^−1^ is the D band corresponding to the disordered *sp*^3^-hybridized carbon atoms of nanotubes, while that at about 1,580 cm^−1^ is the G band corresponding to the structural integrity of the *sp*^2^-hybridized carbon atoms of nanotubes. Thus, the intensity ratio (*I*_D_/*I*_G_) of D to G band can be used to evaluate the extent of defects in the carbon nanotubes. Based on the curves in Figure 4, we found that the intensity ratio of *I*_D_/*I*_G_ was about 1.7 in all cases, which indicated that there was no influence on the structural features of nanotubes before and after the reaction with AETTPy. Besides the D and G bands, there were two weak bands that appeared at 2,660~2,636 and 2,900 cm^−1^, which could be attributed the second-order mode of D and the combination of D and G bands.

For the pythio-MWNT powders and the SAMs of pythio-MWNT nanohybrids, the D and G bands appeared at about 1,333 and 1,587 cm^−1^. This means that both peaks shifted a little (13 cm^−1^) to the higher wavenumbers after functionalization, the feature of which was often observed for the chemical treatment of the CNTs [24]. Besides such a peak shift, no significant difference was observed for the MWNTs before and after functionalization. When the nanotubes reacted with AETTPy and formed SAMs, the Raman spectrum showed several small peaks (Figure 4 (b)) between 200 and 1,500 cm^−1^ as well as a band at 2,885~2,913 cm^−1^. The peak at 251 cm^−1^ was assigned to the Au-S stretch [25,26]. The peaks between 900 and 1,300 cm^−1^ were assigned to the vibration of the C-C stretching vibration coupled to the C-N stretching vibration. The small peak at 1,450 cm^−1^ was assigned to the scissoring mode of the CH_2_ groups present in the functionalized AETTPy. The C-H stretching region of CH_2_ groups showed a prominent band at about 2,855~2,920 cm^−1^ together with the combination of D and G bands of MWNTs.

### Voltammetric properties

**Figure 5:**
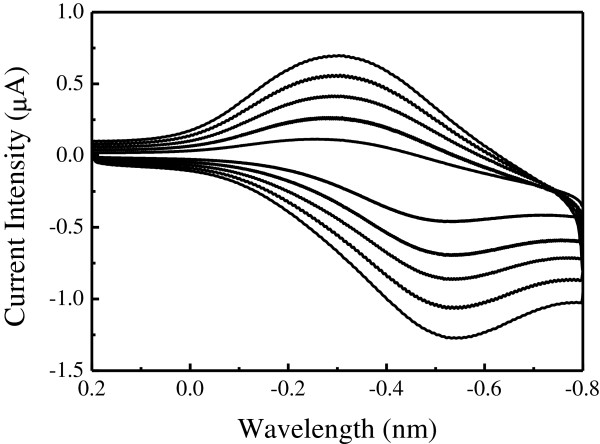
**Cyclic voltammograms.** Gold electrode modified by SAMs of pythio-MWNTs-Cyt c in the 0.01 mol/l KCl electrolyte solutions at the scan rates of 0.1, 0.2, 0.3, 0.4, and 0.5 V/s.

The cyclic voltammograms for the gold electrode covered by the pythio-MWNT-Cyt c nanocomposites were measured in the 10 mmol/l KCl electrolyte solution. A quasi-reversible redox wave was recorded with the cathodic potentials at about −0.55 V and anodic ones at about −0.28 V (vs Ag/AgCl, Figure 5). It has been reported that the cytochrome heme electrochemical midpoint potentials varied between −0.4 and 0.4 V (vs SHE) [27], which was in agreement with the results obtained in the present work. The relative current intensity of the anodic peak was a little weaker than that of the cathodic one, which may be ascribed to the following: (1) the film resistance was increased for the SAM-modified electrode; (2) the distance between the electrode surface and electroactive center of Cyt c was too far, so the electron transfer was inefficient; and (3) the Cyt c may be denaturated on the solid support [27,28]. 

Relation of the redox current intensity of the modified electrode to the scan rate and the root of the scan rate was calculated (curves not shown), which revealed that the current intensity was proportional to the root of the scan rate. This feature suggests that, compared to the diffusion layer, the present pythio-MWNT-Cyt c SAMs was rather thicker. These results are also in agreement with our previous work on the LB films of MWNTs-hydrogenase, wherein it was revealed that, because of the different diameters of nanotubes, the current intensity was proportional to the scan rate for the electrodes modified with the LB films of pure proteins and their composites with single-walled carbon nanotubes, but to the root of scan rate for those modified with the LB films of MWNTs [13]. The redox reaction of Cyt c in the present SAMs was a diffusion control process.

### Morphology characterization

**Figure 6:**
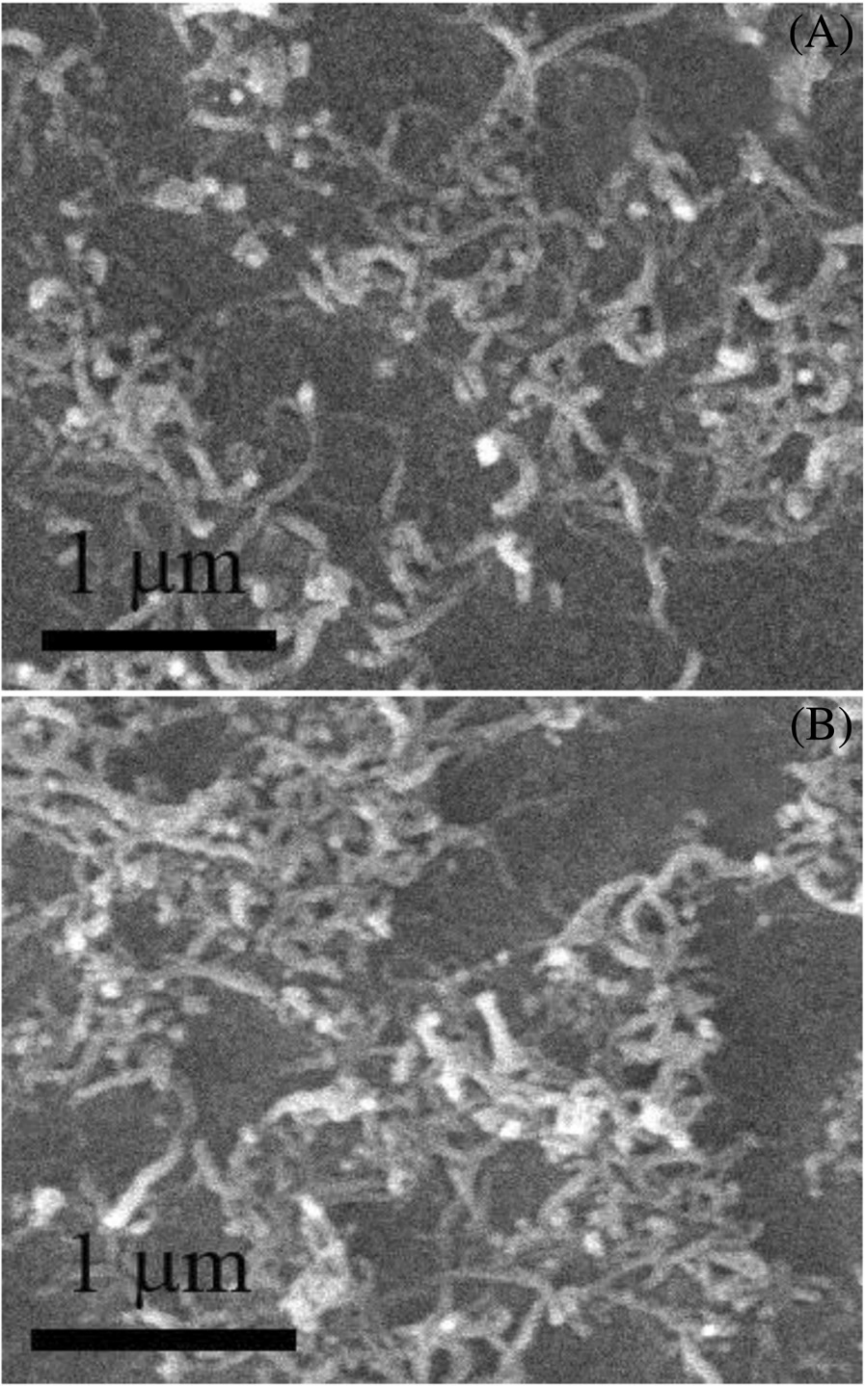
**SEM images for the SAMs of pythio-MWNTs.** (**A**) Before and (**B**) after adsorption of Cyt c.

Morphologies and distribution of the SAMs were characterized using SEM and AFM techniques. These SAMs were prepared on the gold surface, which were then immersed in the aqueous solution of Cyt c at room temperature. Figure 6 shows the SEM images for the SAMs of pythio-MWNTs before and after adsorption of Cyt c, which revealed the following features.

Firstly, the functionalized nanotubes formed an irregular densely packed monolayer in the SAMs (Figure 6A), which was similar to that of the pythio-MWNT LB films deposited at about 20 mN/m [17]. This image provided a direct evidence for the formation of SAMs of the nanotubes. Secondly, after the SAMs were immersed in the solution of Cyt c, more dense and larger aggregates contained in nanotubes were observed in the 2D SEM image (Figure 6B), which may be attributed to the reason that the protein was adsorbed on the pythio-MWNT SAMs.

It was revealed that the casting film of Cyt c formed irregular distribution of the protein aggregates separated on the substrate surface, which was much different from that adsorbed on the present SAMs. This difference may be attributed to the fact that the molecular interaction was different between the Cyt c and pythio-MWNTs from that between the protein and Si surface. Based on literatures, it has been concluded that electrostatic interaction and van der Waals or hydrophobic interaction between alkyl chains of amphiphiles and the sidewalls of CNTs, as well as the protein-CNT affinity, play important roles in the formation of CNT-protein conjugates [29]. Here, because the -COOH groups in the oxidized MWNTs have connected with AETTPy, the hydrophobic interaction and protein affinity between Cyt c and pythio-MWNTs dominated the protein adsorption on the pythio-MWNTs [30]. For the casting films, the physical adsorption (van der Waals interaction) dominated aggregates of proteins.

**Figure 7:**
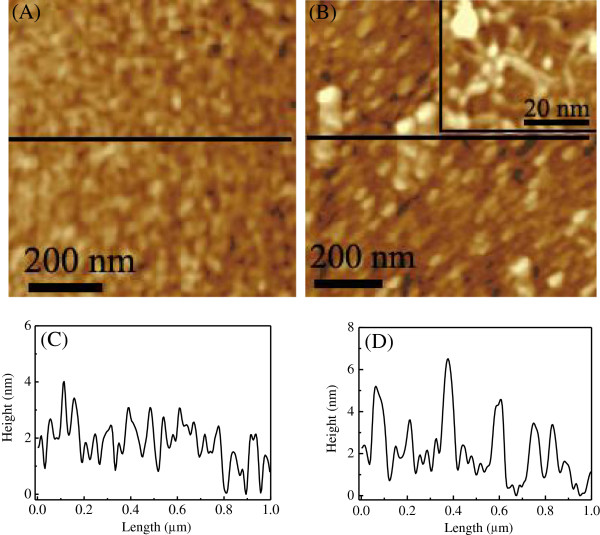
**AFM images for the SAMs of pythio-MWNTs.** (**A**) Before and (**B**) after adsorption of Cyt c. (**C**, **D**) Height profiles corresponding to the lines in the AFM images of (**A**) and (**B**), respectively.

To give more microscopic information on the SAMs of pythio-MWNTs, their topographic images and height distribution before and after the adsorption of Cyt c were investigated by AFM. As shown in Figure 7A, irregular pythio-MWNT aggregates were observed for the SAMs before immersion in the Cyt c. After the SAMs were immersed in the Cyt c solution for 1 h, dot-like aggregates could be distinguished from the AFM image, with the sizes of the aggregates increased (Figure 7B). These aggregates could be attributed to the Cyt c adsorbed on the surface of pythio-MWNTs. A higher resolution AFM photo was inserted in Figure 7B, from which tubular lines of the nanotubes could be observed.

The height profiles obtained from the AFM images were shown in Figure 7C,D. These curves indicated that the height of most aggregates in the SAMs of pythio-MWNTs was around 3 nm. When the protein was adsorbed on the SAMs, the average height of the aggregates increased, with some domains reaching as high as 6 nm. These data further confirmed that the Cyt c was adsorbed on the surface of pythio-MWNTs.

## Conclusions

We have demonstrated preparation of the self-assembled monolayers of pyridylthio-functionalized multiwalled carbon nanotubes on the gold substrate surface, which could be used as a support to immobilize cytochrome c to form bio-nanocomposites. The surface coverage for the SAMs of pythio-MWNTs was about 5.2 μg/cm^2^ and that of the Cyt c was about 0.29 μg/cm^2^. It was suggested that the protein was adsorbed on the surface of the nanotubes through the hydrophobic interaction and protein affinity between the Cyt c and pythio-MWNTs.

## Competing interests

The authors declare that they have no competing interests.

## Authors' contributions

QS and JL carried out the synthesis and characterizations of the materials and drafted the manuscript. HXH carried out the Raman spectroscopy and electrochemistry. MC and DJQ contributed to the design and discussion of this work and in the revision of the manuscript. All authors read and approved the final manuscript.
